# Angle Class I malocclusion with anterior negative overjet

**DOI:** 10.1590/2177-6709.21.2.102-114.bbo

**Published:** 2016

**Authors:** Paulo Ávila de Souza

**Affiliations:** 1 Specialist in Orthodontics, Pontifícia Universidade Católica de Minas Gerais (PUC-MG), Belo Horizonte, Minas Gerais, Brazil. Diplomate of the Brazilian Board of Orthodontics and Facial Orthopedics (BBO).

**Keywords:** Angle Class I malocclusion, Orthopedics, Corrective Orthodontics.

## Abstract

This clinical case report describes the orthodontic treatment of an 8-year and 9-month old female patient with Angle Class I malocclusion, anterior crossbite and canine Class III relationship. Orthodontic treatment was carried out in two stages. The first one was orthopedic, while the second one included the use of a fixed appliance and the need for space gain for reshaping of maxillary lateral incisors. The two-stage treatment combined with multidisciplinary Restorative Cosmetic Dentistry allowed excellent esthetic and functional outcomes to be achieved. This case was presented to the Brazilian Board of Orthodontics and Dentofacial Orthopedics (BBO) as a requirement for the title of certified by the BBO.

## RESUMO

O presente caso clínico relata o tratamento ortodôntico de uma paciente com oito anos e nove meses de idade, portadora de má oclusão de Classe I de Angle, com mordida cruzada anterior e relação de classe III entre os caninos. O tratamento ortodôntico foi realizado em duas etapas, sendo a primeira ortopédica e a segunda constando de aparatologia fixa, havendo necessidade de ganho de espaço para reanatomização dos incisivos laterais superiores. O tratamento em duas etapas, aliado à multidisciplinariedade com a Dentística Restauradora, permitiu a obtenção de excelente resultado final estético e funcional. O presente caso clínico foi apresentado à Diretoria do Board Brasileiro de Ortodontia e Ortopedia Facial (BBO) como parte dos requisitos para obtenção do título de Diplomado pelo BBO.

## INTRODUCTION

A Caucasian, 8-year and 9-month old, female patient in good general oral health was referred for treatment by her legal guardians, with the major esthetic complaint of anterior negative overjet. She had no functional complaint. The patient's mother and two sisters had Class III skeletal pattern, which revealed a strong possibility of her having the same unfavorable direction of facial growth.

## DIAGNOSIS

**Figure 1:**
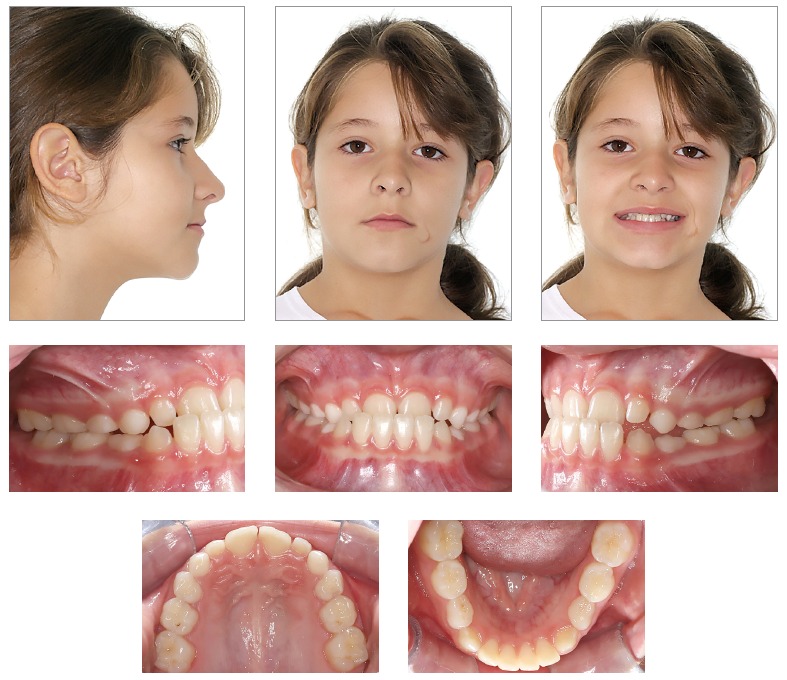
Initial facial and intraoral photographs.

**Figure 2:**
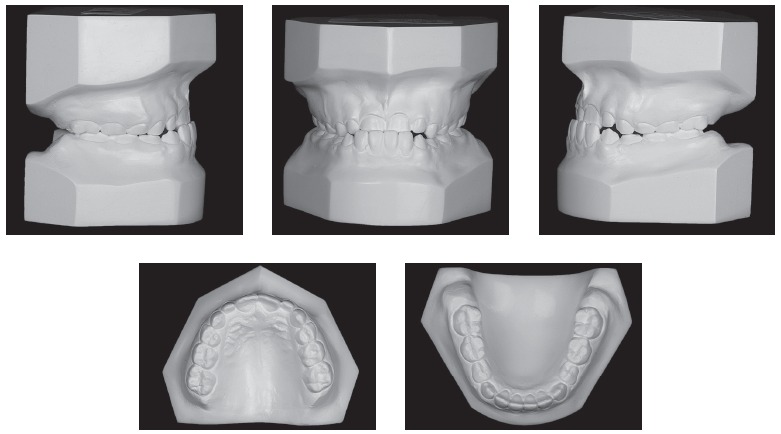
Initial casts.

Facial analysis in frontal view revealed the presence of passive lip seal. At smiling, she had little maxillary incisors exposure, with discreet asymmetry of the mandible to the right. In lateral view, the patient presented with a deficiency in the premaxilla and a concave profile with a tendency to become worse overtime.^1,2,3^ There was more lower lip protrusion in comparison to the upper lip, with an open nasolabial angle. The aforementioned features are shown in Figure 1. Dental assessment (Figs 1, 2) revealed molars in Class I relationship^4^ and canines in Class III relationship, with crossbite in the anterior region. Patient's mandibular midline had a 1-mm shift to the right, while maxillary lateral incisors had crowns significantly reduced in size.

**Figure 3:**
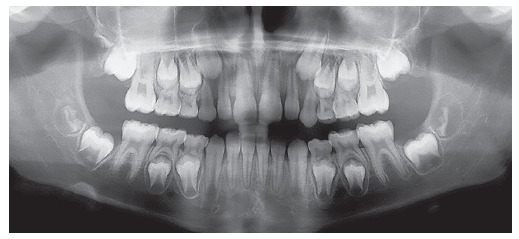
Initial panoramic radiograph.

Panoramic radiograph (Fig 3) revealed the presence of all permanent teeth at different odontogenic stages, with teeth #18, #38 and #48 found to be at the initial stages of crown formation.

**Figure 4:**
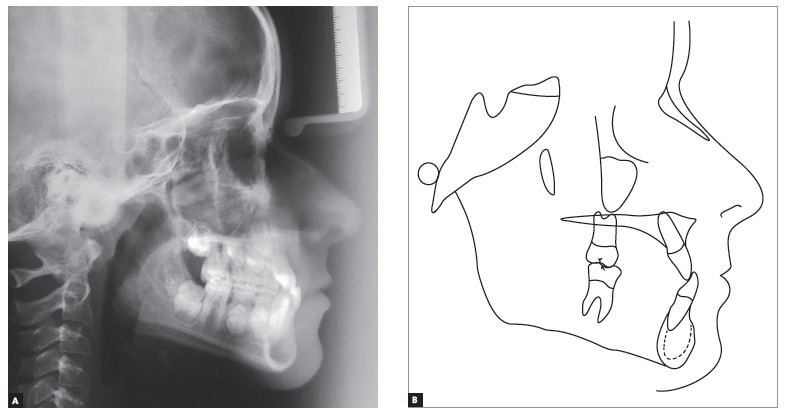
Initial lateral cephalogram (A) and cephalometric tracing (B).

Cephalometric analysis (Fig 4) revealed a Class I skeletal pattern (ANB = 1°). Despite high SNA and SNB angle values (93° and 92°, respectively), both maxilla and mandible were well positioned in relation to the base of the skull. Patient's lower facial height was decreased in vertical direction (SN-GoGn = 20° and FMA = 14°), with some tendency towards labial protrusion of mandibular incisors (IMPA = 95°). The aforementioned cephalometric data are shown in Table 1.

**Table 1:** Initial cephalometric values (A) at the beginning of the second treatment phase (A1) and final (B) cephalometric values.

	Measurements		Normal	A	A1	B	Dif. A/B
Skeletal pattern	SNA	(Steiner)	82°	93°	93°	92°	1
	SNB	(Steiner)	80°	92°	91°	90°	2
	ANB	(Steiner)	2°	1°	2°	2°	1
	Wits	(Jacobson)	♀ 0 ±2 mm ♂ 1 ±2 mm	-2 mm	-3 mm	-2 mm	0
	Angle of convexity	(Downs)	0°	3°	4°	2.5°	0.5
	Y-axis	(Downs)	59°	55°	59°	61.5°	6.5
	Facial angle	(Downs)	87°	95°	96°	96°	1
	SN-GoGn	(Steiner)	32°	20°	20°	22°	2
	FMA	(Tweed)	25°	14°	16°	17°	3
Dental pattern	IMPA	(Tweed)	90°	95°	95°	94°	1
	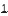 .NA (degrees)	(Steiner)	22°	16°	19°	21°	5
	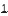 -NA (mm)	(Steiner)	4 mm	1 mm	4 mm	5 mm	4
	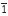 .NB (degrees)	(Steiner)	25°	28°	28°	26°	2
	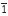 -NB (mm)	(Steiner)	4 mm	4 mm	4 mm	4 mm	0
	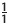 - Interincisal angle	(Downs)	130°	136°	131°	132°	4
	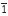 -APo	(Ricketts)	1 mm	3 mm	3 mm	3 mm	0
Profile	Upper lip - S-line	(Steiner)	0 mm	-1.5 mm	-1.5 mm	-3 mm	1.5
	Lower lip - S-line	(Steiner)	0 mm	-0.5 mm	-3 mm	-3.5 mm	3

## TREATMENT PLAN

In view of patient's conditions, a two-stage treatment plan was established: the first stage would include orthopedic intervention, while the second one would include the use of a mandibular fixed tongue crib followed by conventional orthodontic treatment.

Initially, a progenic appliance with digital springs would be placed in order to protrude maxillary incisors, which would correct anterior crossbite. Subsequently, the patient would be followed-up, so as to have the development of her dentition monitored. Immediately before her mandibular deciduous second molars were lost, she would have a mandibular fixed tongue crib placed in order to have the leeway space^5^ preserved and the action of the tongue minimized, thereby leading to a physiological retraction of mandibular incisors. Thereafter, orthodontic bands would be installed on teeth #16 and #26 and brackets (MBT Straight Wire slot 0.022 x 0.028-in) would be bonded to all other teeth in both upper and lower arches.

For alignment and leveling, stainless steel Twist Flex 0.015-in, 0.0175-in and 0.020-in archwires, followed by 0.016-in and 0.018-in smooth archwires and 0.018 x 0.025-in rectangular wires would be used. In the maxilla, the spaces between mesial and distal surfaces of lateral incisors would be preserved, so as to allow resin to be placed in those areas; thus, improving the anatomical traits.

For the retention phase, treatment plan included the use of a 1.5-mm acetate sheet in the maxilla, and an intercanine bar manufactured with stainless steel Twist Flex 0.032-in wire in the mandible.

## TREATMENT PROGRESS

**Figure 5:**
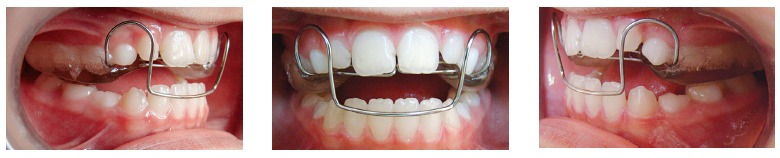
Protraction archwire.

Treatment was carried out as planned without changes in the planned sequence. Initially, an orthopedic progenic appliance with digital springs was installed in the anterior region, aiming at protruding maxillary incisors (Fig 5). After anterior crossbite had been corrected, a maxillary removable bite plate was used to favor extrusion of posterior mandibular teeth, thus correcting posterior open bite caused during crossbite correction.

**Figure 6:**
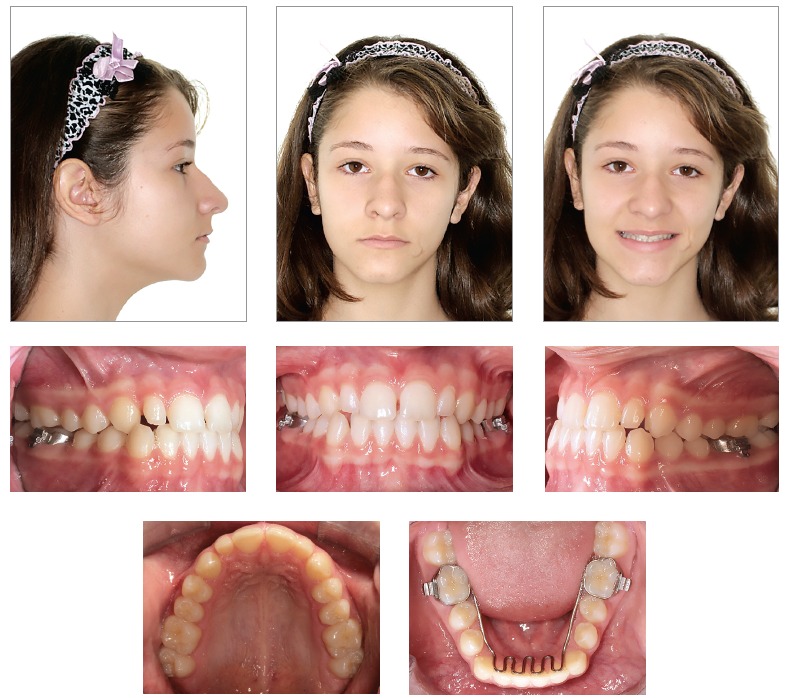
Intermediate facial and intraoral photographs.

**Figure 7:**
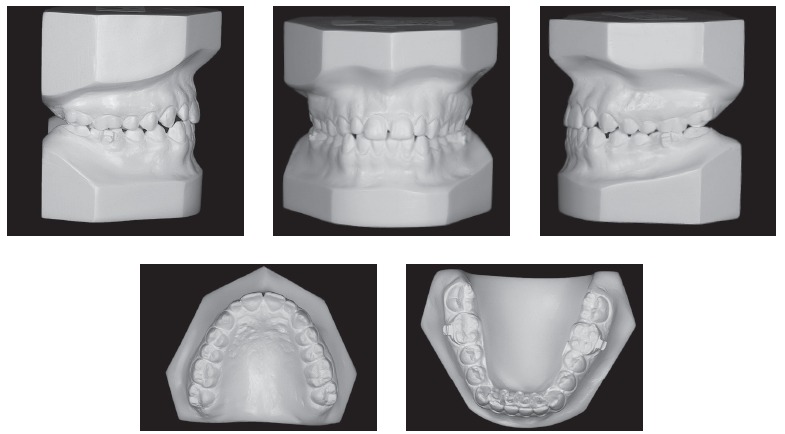
Intermediate casts.

**Figure 8:**
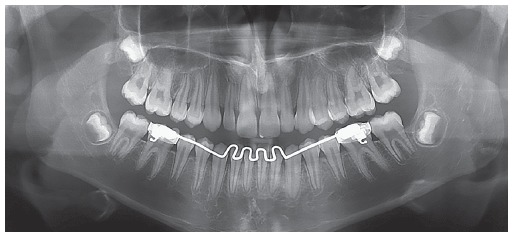
Intermediate panoramic radiograph.

**Figure 9:**
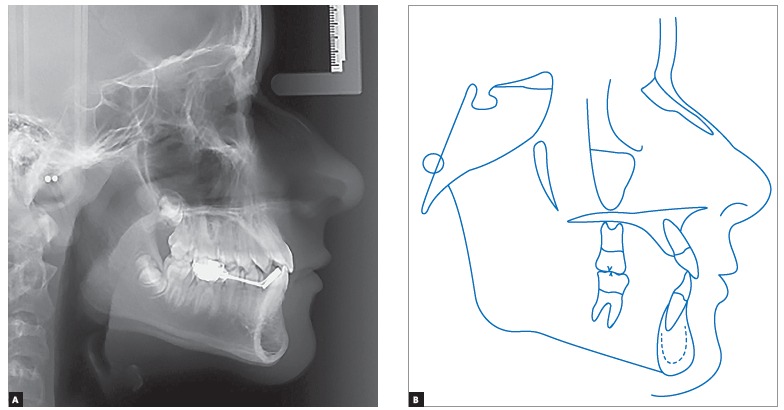
Intermediate lateral cephalogram (A) and cephalometric tracing (B).

The patient was followed-up and before exfoliation of mandibular deciduous second molars, a fixed tongue crib was installed, supported by teeth #36 and #46. At 11 years and nine months of age, after mandibular second premolars had fully erupted, and the patient had complete permanent dentition, except for third molars, new examination was required with a view to initiating conventional orthodontic treatment (Figs 6 to 9).

Orthodontic bands were placed around maxillary permanent first molars and brackets bonded to all other maxillary and mandibular teeth. Metal brackets (MBT Straight Wire, slot 0.022 x 0.018-in) were used.

Alignment and leveling archwires were placed in the maxilla and mandible in the following sequence: Twist Flex stainless steel 0.015-in, 0.0175-in and 0.020-in wires, stainless steel smooth 0.016-in and 0.018-in round wires, and stainless steel 0.018 x 0.025-in rectangular wires. With a view to gaining space adjacent to the mesial and distal surfaces of lateral incisors in the upper arch, open springs were compressed between central incisors and canines. The space was used for reshaping of lateral incisors carried out by means of placing resin in the proximal surface of those teeth.

Once the 0.018-in steel wire had been installed, an elastomeric chain was placed from tooth #36 to #46 in the lower arch, combined with intermaxillary Class III elastics, with a view to not only closing residual spaces resulting from leeway space, from posterior to anterior direction up to the region of incisors, but also to enhance retraction of those teeth. At this stage, the fixed tongue crib was removed and spaces fully closed.

Subsequently, the finishing phase began. To this end, new stainless steel 0.018 x 0.025-in archwires were placed in both upper and lower arches, with customized bends and torques, as necessary. With a view to adjusting intercuspation between maxillary and mandibular teeth, the segmented arch technique was employed and intermaxillary elastics were placed.

Once all treatment goals had been achieved, the orthodontic fixed appliance was removed and the retention phase started. A removable appliance manufactured with a 1.5-mm acetate sheet was used in the maxilla, while an intercanine bar manufactured with stainless steel Twist Flex 0.032-in wire was used in the mandible.

## RESULTS

**Figure 10:**
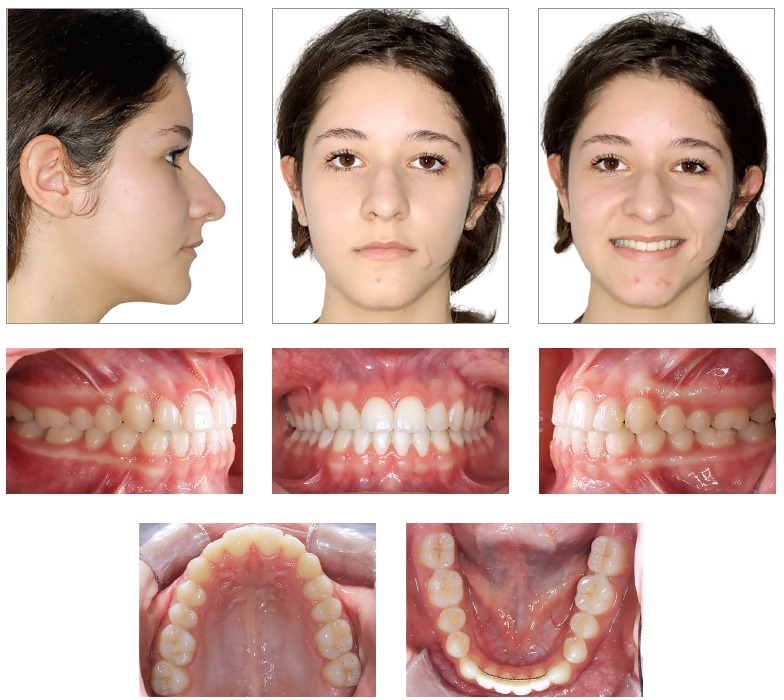
Final facial and intraoral photographs.

**Figure 11:**
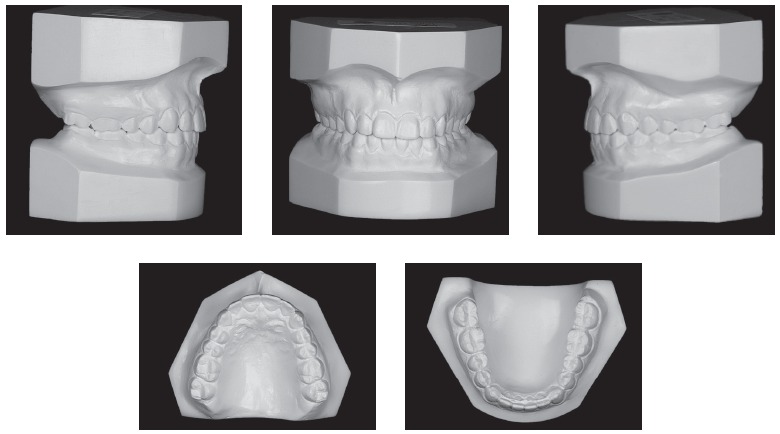
Final casts.

**Figure 12:**
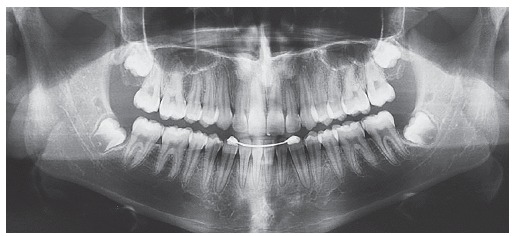
Final panoramic radiograph.

**Figure 13:**
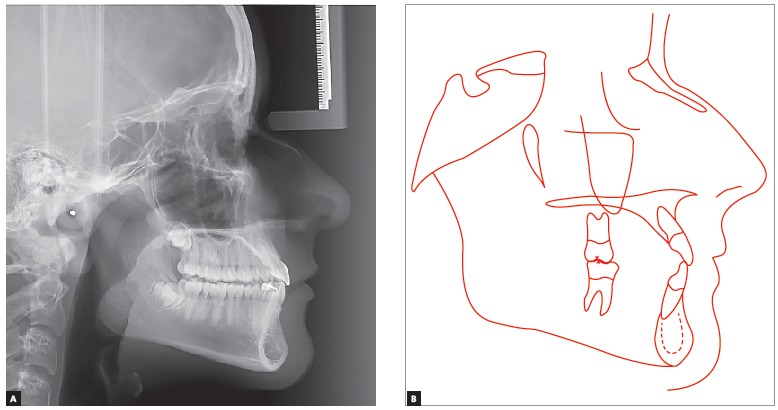
Final lateral cephalogram (A) and cephalometric tracing (B).

Patient's final records (Figs 10 to 13) assessment revealed that all treatment goals were achieved. There was vertical gain in patient's lower third of the face, in addition to significant upper lip protrusion, thereby improving patient's profile significantly. However, passive lip seal was preserved. Moreover, patient's smile was significantly improved, with greater maxillary incisors exposure.

Dental assessment revealed canines and molars in Class I relationship on both sides, in addition to coinciding maxillary and mandibular midlines and correction of both overbite and overjet. Functional harmony was excellent for occlusion in protrusive excursion and right as well as left lateral guidance, with centric relation coinciding with maximal intercuspation. It is worth noting that, as shown by panoramic radiograph taken at treatment completion (Fig 12), changes were achieved without radiographically noticeable apical root remodeling.

As planned, cephalometric examination revealed that patient's skeletal pattern was preserved, with the ANB^6^ angle increasing from 1° to 2°, and Wits value remaining unchanged at -2 mm. There was an increase in lower facial height (SN-GoGn increased from 20° to 22° while FMA increased from 14° to 17°). The Y-axis angle increased from 55° to 61.5°, revealing vertical clockwise mandibular rotation, thereby compensating Class III. These data are shown in Figure 13 and Table 1.

**Figure 14:**
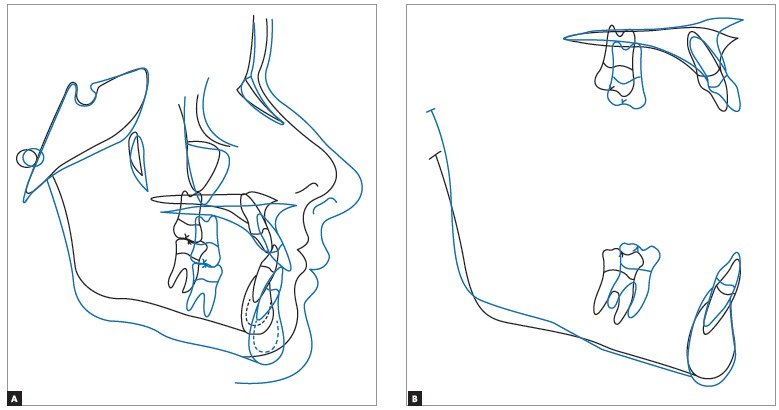
Total (A) and partial (B) cephalometric superimpositions of initial (black) and second treatment phase completion (blue) tracings.

Cephalometric superimposition (Fig 14) revealed maxillary incisors protrusion and vertical gain with clockwise mandibular rotation; thus, providing the patient with a significantly improved facial profile.

**Figure 15:**
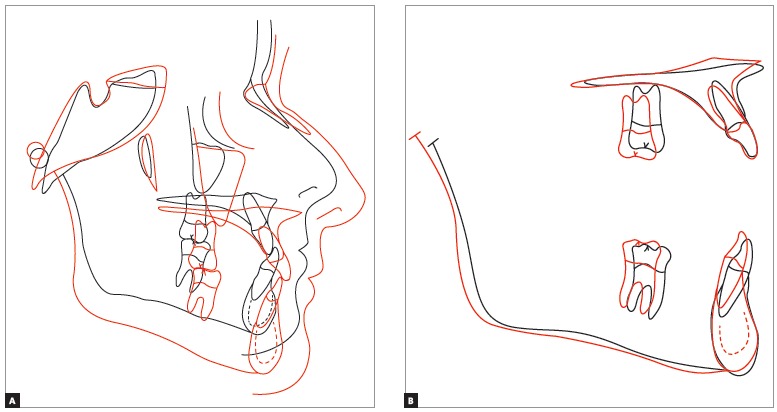
Total (A) and partial (B) cephalometric superimpositions of initial (black) and final (red) tracings.

## FINAL CONSIDERATIONS

The role played by heredity in the etiology of Class III malocclusion has been increasingly reported, since the skeletal component is often compromised. For this reason, the yielded results cannot be definitive, although several authors have reported the potential for success achieved by conventional therapy,^8^ which warrants early treatment of such malocclusion.

As it has been previously reported, there was some concern about proportionality between maxillary central and lateral incisors dimensions. Since lateral incisors were rather small, there was a need for space gain in their mesial and distal surfaces, for future improvements with composite resin. Interdisciplinary action was key to balance the dimensions of anterior teeth, thus providing the patient with a much more esthetically pleasing smile.
